# Uncommon complications arising during endoscopic ultrasound-guided gastroenterostomy – splenic injury

**DOI:** 10.1055/a-2603-7497

**Published:** 2025-06-18

**Authors:** Shan-Shan Hu, Peng Tang, Jie Hou, Yun-Chao Yang, Wei-Hui Liu

**Affiliations:** 1 89669 Department of Gastroenterology and Hepatology, Sichuan Provincial Peopleʼs Hospital, School of Medicine, University of Electronic Science and Technology of China, Chengdu, Sichuan Province, China

Endoscopic ultrasound (EUS)-guided gastroenterostomy (EUS-GE) is an advanced technique primarily used to treat benign and malignant gastric outlet obstruction 1 2 . This technology overcomes the limitations of traditional duodenal stent placement and surgical procedures 3 , offering an effective alternative therapy. Although the one-step delivery system significantly reduces procedural risks, adverse events still occur. Common complications are often related to stent displacement or blockage 4 5 . This article reports a rare adverse event – spleen injury.

A 65-year-old female patient, with duodenal cancer causing gastric outlet obstruction and accompanying systemic metastasis, was no longer a candidate for surgical resection ( Fig. 1 ). Therefore, the patient was referred to EUS-GE to alleviate the obstruction. EUS and X-rays were used to locate the target intestine ( Fig. 2 ). After identifying a suitable location for the gastroenterostomy, direct puncture was made with a 15-mm cautery-enhanced lumen-apposing metal stent (LAMS) from the gastric wall into the jejunum. However, due to the transverse shape and frequent peristalsis of the target intestine, the puncture space was limited, causing the tip of the LAMS delivery system to penetrate the opposite intestinal wall and mistakenly enter the spleen at the distal jejunum ( Fig. 3 ). Despite successful stent release ( Fig. 4 ), the patient experienced severe left abdominal pain postoperatively. Subsequent abdominal CT scans revealed a subcapsular splenic hematoma and intraperitoneal hemorrhage. Given the patient’s hemodynamically stable condition, observation and conservative treatment were pursued, and the condition ultimately stabilized ( Fig. 5 ). The patient was successfully discharged. One month post-surgery, the spleen’s shape had returned to normal ( Video 1 ).

**Fig. 1:**
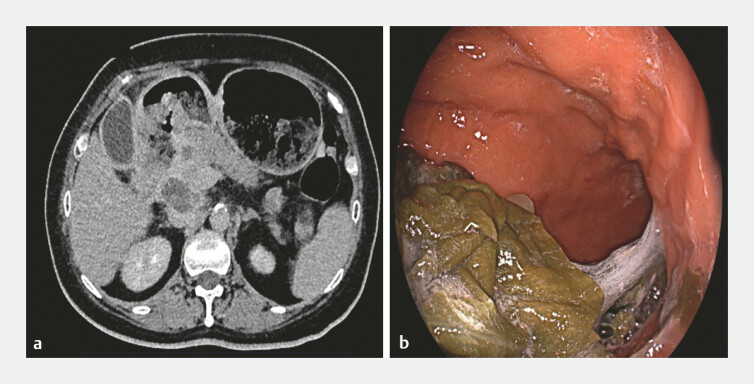
Duodenal cancer leads to gastric outlet obstruction.

**Fig. 2:**
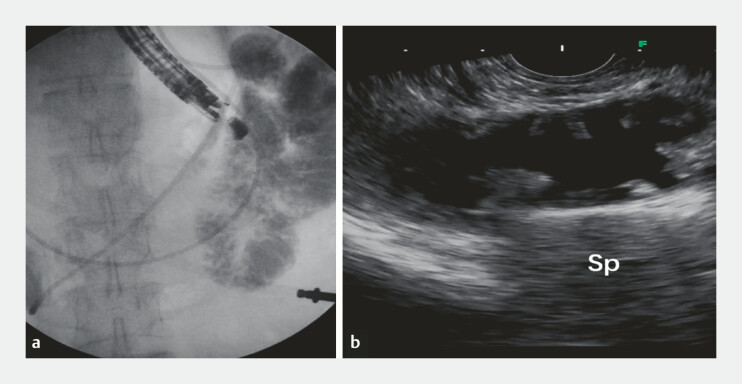
EUS and X-rays were used to select the target intestine. **a** The intestine closest to the gastric wall and jejunum was selected under X-rays, ensuring no stricture in the distal intestine. **b** The morphology of the target intestine observed under EUS was transverse. Abbreviation: EUS, endoscopic ultrasound.

**Fig. 3:**
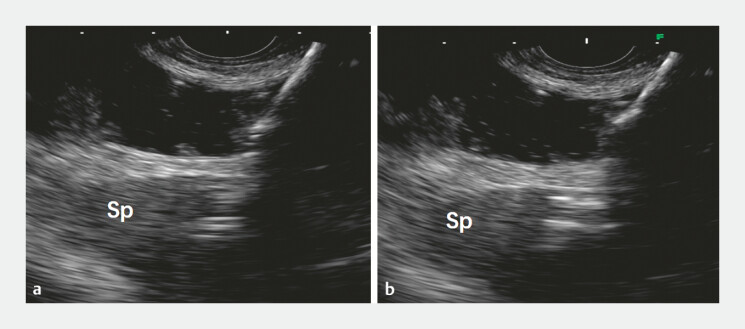
EUS images revealed damage to the spleen caused by the puncture needle. ( **a** ) The tip of the puncture needle penetrated into the spleen. ( **b** ) The high-echo gas shadow inside the spleen after the needle tip was retracted. Abbreviation: EUS, endoscopic ultrasound.

**Fig. 4:**
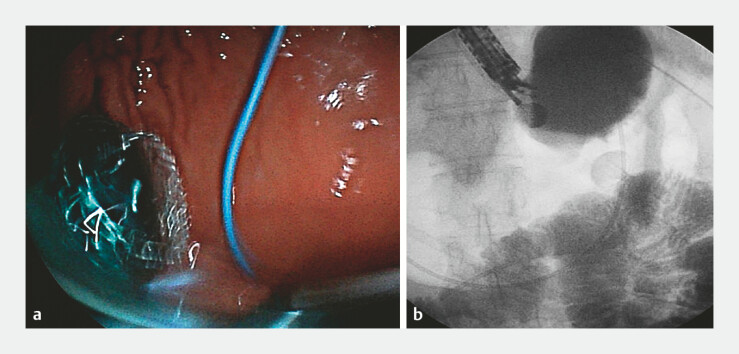
The release position of the stent was ideal. **a** Methylene blue dye flowed smoothly from the jejunum into the stomach cavity through the stent. **b** The exact position of the stent under X-ray.

**Fig. 5:**
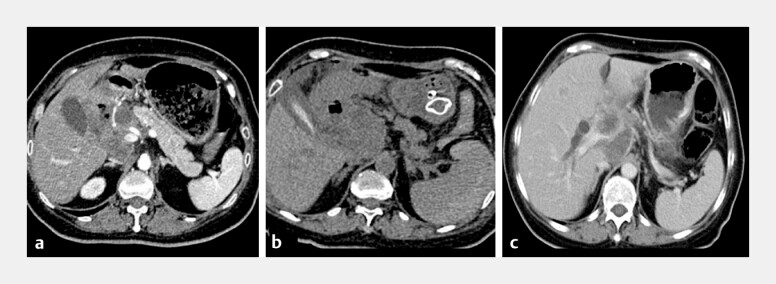
Comparison of preoperative and postoperative CT imaging of the spleen. **a** Normal splenic morphology prior to surgery. **b** Postoperative splenic swelling, subcapsular hematoma, and intraperitoneal hemorrhage. **c** One month post-surgery, the spleen’s shape had returned to normal. Abbreviation: CT, computed tomography.

Uncommon complications arising during endoscopic ultrasound-guided gastroenterostomy – splenic injury.Video 1

From this case, we learned that, it is important to choose a loop that appears longitudinal under EUS to provide sufficient space for puncture and stent release. Before puncturing, intestinal peristalsis should be minimized, and careful observation of the surrounding organs is necessary. During the puncture, continuous attention should be given to the position of the needle tip to ensure the safety of the procedure.

Endoscopy_UCTN_Code_TTT_1AS_2AI

**Correction**

**Correction: Uncommon complications arising during endoscopic ultrasound-guided gastroenterostomy – splenic injury** Hu Shan-Shan,Tang Peng, Hou Jie et al. Uncommon complications arising during endoscopic ultrasound-guided gastroenterostomy – splenic injury. Endoscopy 2025; 57: E595–E597, doi:10.1055/a-2603-7497 In the original published version of this article, the video was incorrect. The video has been replaced with the correct version on July 22, 2025.

## Notes

Endoscopy E-Videos https://eref.thieme.de/e-videos
*E-Videos* is an open access online section of the journal *Endoscopy* , reporting on interesting cases and new techniques in gastroenterological endoscopy. All papers include a high-quality video and are published with a Creative Commons CC-BY license. Endoscopy E-Videos qualify for HINARI discounts and waivers and eligibility is automatically checked during the submission process. We grant 100% waivers to articles whose corresponding authors are based in Group A countries and 50% waivers to those who are based in Group B countries as classified by Research4Life (see: https://www.research4life.org/access/eligibility/ ). This section has its own submission website at https://mc.manuscriptcentral.com/e-videos .

## Notes

Correction **Correction: Uncommon complications arising during endoscopic ultrasound-guided gastroenterostomy – splenic injury** Hu Shan-Shan,Tang Peng, Hou Jie et al. Uncommon complications arising during endoscopic ultrasound-guided gastroenterostomy – splenic injury. Endoscopy 2025; 57: E595–E597, doi:10.1055/a-2603-7497 In the original published version of this article, the video was incorrect. The video has been replaced with the correct version on July 22, 2025.
