# Correction: Uncommon complications arising during endoscopic ultrasound-guided gastroenterostomy – splenic injury

**DOI:** 10.1055/a-2649-2201

**Published:** 2025-07-22

**Authors:** Shan-Shan Hu, Peng Tang, Jie Hou, Yun-Chao Yang, Wei-Hui Liu

**Affiliations:** 1 89669 Department of Gastroenterology and Hepatology, Sichuan Provincial Peopleʼs Hospital, School of Medicine, University of Electronic Science and Technology of China, Chengdu, Sichuan Province, China



In the original published version of this article, the video was incorrect. The video has been replaced with the correct version on July 22, 2025.

